# HAPPY MAMA Project (Part 2)—Maternal Distress and Self-Efficacy: A Pilot Randomized Controlled Field Trial

**DOI:** 10.3390/ijerph19031461

**Published:** 2022-01-27

**Authors:** Alice Mannocci, Sara Ciavardini, Federica Mattioli, Azzurra Massimi, Valeria D’Egidio, Lorenza Lia, Franca Scaglietta, Andrea Giannini, Roberta Antico, Barbara Dorelli, Alessandro Svelato, Luigi Orfeo, Pierluigi Benedetti Panici, Antonio Ragusa, Giuseppe La Torre, HAPPY MAMA Group

**Affiliations:** 1 Faculty of Economics, Mercatorum University, 00186 Rome, Italy; 2 Department of Public Health and Infectious Diseases, Sapienza University, 00185 Rome, Italy; saraciavardini@gmail.com (S.C.); mattioli.federica@aslto5.piemonte.it (F.M.); azzurra.massimi@uniroma1.it (A.M.); v.degidio@sanita.it (V.D.); lorenza.lia@uniroma1.it (L.L.); f.scaglietta@gmail.com (F.S.); Robantico76@gmail.com (R.A.); barbara.dorelli@uniroma1.it (B.D.); giuseppe.latorre@uniroma1.it (G.L.T.); alice.mannocci@uniroma1.it (H.M.G.); 3 Department of Maternal and Child Health and Urological Sciences, Umberto I Teaching Hospital, Sapienza University, 00185 Rome, Italy; andrea.giannini@uniroma1.it (A.G.); pierluigi.benedettipanici@uniroma1.it (P.B.P.); 4 Department of Obstetrics and Gynecology, San Giovanni Calibita Fatebenefratelli Hospital, 00186 Rome, Italy; alessandrosvelato@virgilio.it (A.S.); antonio.ragusa@fbf-isola.it (A.R.); 5 Neonatal Intensive Care Unit, “San Giovanni Calibita” Fatebenefratelli, 00186 Rome, Italy; luigi.orfeo@fbf-isola.it

**Keywords:** self-efficacy, mindfulness, stress, post-partum, newborn, mother-infant, maternal behavior, mother-infant interaction, maternal parenting stress, maternal support

## Abstract

Introduction: The aim of the pilot randomized controlled field trial is to assess if a midwifery intervention is able to increase the maternal self-efficacy and reduce the stress level during the first six months after birth. Methods: The study was conducted in two different hospitals in Rome, Italy, involving women delivering at or beyond term, aged >18 years old and with normal APGAR scores of the infant. The participants were randomly divided into two groups: “*Individual Intervention Group*” (they received home midwifery assistance for one month after birth, I) and the “*Control Group*” (C). A self-administered questionnaire was administered four times: at the baseline about one week after the hospital delivery (T0), after the intervention about one month after the delivery (T1), and at three months (T2) and at six months after birth (T3). The questionnaire included different validated scales needed to assess maternal perceived self-efficacy (KPCS), parental stress scale stress (PSS) and maternal depressive risk symptoms (EPDS). Results: The study population counted 51 mothers: 28 women in the “C” group and 23 women in the “I” group. The PSS score was statistically higher in the “C” than “I” group at T1 (*p* = 0.024); whereas the KPCS score was statistically higher in the “I” (*p* = 0.039) group; EPDS score did not show significant difference between the two groups in the follow-up period. An inverse significant correlation between KPCS and PSS was found during the study window time (*p* < 0.0001). Conclusions: These results potentially give the opportunity to explore this area of focus further, in order to better address maternal individual needs for the successful transition to motherhood. More research in this area is required.

## 1. Introduction

In spite of the popular saying that women have a maternal instinct, the postpartum period is a time very commonly accompanied by anxiety, uncertainties, and fear [1]. Women have to quickly adapt to a new routine and face new responsibilities and tasks, such as breastfeeding, which can be challenging for a primiparous mother. The period after birth is often characterized by changes in sleeping habits, family dynamics, but also changes in the woman’s body, and as the presence of several physical health conditions, have been found in the two years postpartum [1,2,3]. These can cause distress and fatigue in a new mother, which can be aggravated in case of lack of social support and a sound financial condition [4].

Until now, research on this topic has focused on postpartum depression [5] while postpartum maternal health was often neglected [6,7]. Considering that the stress and other psychological conditions during this period can have negative consequences for the mother and the baby [8], research in the area of psychological suffering during the period after birth is needed.

But why should a new mother be stressed out? This may occur when a new mother perceives that responsibilities exceed her available coping resources, and thus she will experience stress [9], and chronic stress can result in mental health problems [10].

For this reason, it should be taken into consideration that during the postpartum period, the motherhood can be supported by health interventions that give support and information [8,11].

For assessing the efficacy of the interventions that focused on the increase of wellbeing and reduction of anxiety, stress and depression should be monitored. Mindfulness and self-efficacy [12] are considered key elements in influencing pain intensity, daily functioning, decision-making and the ability to self-regulate and control personal destiny [13].

What are self-efficacy and mindfulness in motherhood?

Self-efficacy refers to an individual’s’ beliefs in their ability to successfully perform specific behaviors needed to function effectively in a particular domain [14,15,16]. When contextualizing self-efficacy in the parental domain, it is referred to as maternal parental self-efficacy (MPSE) [17]. The MPSE encompasses both the level of perceived knowledge of appropriate child-rearing behaviors and the degree of confidence in one’s ability to perform parenting tasks. For a mother to perceive herself as efficacious in parenting, she must have (a) a repertoire of responses to typical child-rearing situations (e.g., methods of soothing a crying infant, ways to manage a toddler’s disruptive behavior), (b) confidence in her ability to carry out these interventions, (c) beliefs that her child will respond to her efforts, and (d) beliefs that significant others will support her efforts [18,19]. It is the cognitive belief mothers hold in their ability to perform newborn-care tasks, and it is one of the most crucial components for the smooth transition to motherhood [20,21,22].

Mindfulness is the awareness that emerges when we learn to pay deliberate and wholehearted attention to the moment-by-moment unfolding of the external and internal world [23]. Consequently, the mindfulness-based interventions (MBIs) have been identified as stress-reducing and psychological improvement enhancers [24]. In the context of pregnancy and motherhood, a recent systematic review and meta-analysis of mindfulness-based interventions in pregnancy found that mindfulness-based interventions may be beneficial for outcomes such as anxiety, depression, perceived stress and levels of mindfulness [25]. Similarly, a systematic review of the effectiveness of MBIs on maternal perinatal mental health outcomes offered preliminary evidence for the effectiveness of the interventions in reducing anxiety [26].

A pilot study showed that mothers that received a MBIs in the perinatal and postpartum period reported significantly higher maternal self-efficacy, mindfulness components, and self-compassion than those in the control group, and also reported lower anxiety, stress, and psychological distress [27].

Another qualitative study has revealed that women often feel the need for help after delivery [28]. Evidence from Finland has shown that breastfeeding is eased and continued due to the mother’s resources and attitude to breastfeeding, support from her social network, and the current promotion of breastfeeding in society [29] which could be increased by interventions that give support and information to women.

The purpose of the present pilot study is to assess if an intervention focused to increase the maternal well-being and self-efficacy of Italian women during the first six months after childbirth prevents or reduces stress levels and the risk of depression. The main hypothesis of the study is that women with higher level of maternal self-efficacy experience lower levels of stress.

This study aims to engage and empower new mothers by strengthening their parenting skills. High parental confidence predicts several parental and child outcomes [30] and act as a protective factor against maternal depression, stress, relationship difficulties and compromised child development [1]. The conceptual framework underlying the intervention is Bandura’s self-efficacy theory: self-efficacy is defined as “the belief in one’s capabilities to achieve a goal or perform a task and can influence personal motivation and ability to succeed” [15,31]. This approach was widely used for health promotion and to obtain behavioral changes in several contexts and in different kinds of patients. In this specific context, parental self-efficacy is defined as “the beliefs or judgments a parent holds of their capabilities to organize and execute a set of tasks related to parenting a child’’ [32]. Parental efficacy could also be defined as “the parent’s beliefs in his or her ability to influence the child and his or her environment to foster the child’s development and success” [14].

Therefore, the HAPPY MAMA intervention is globally focused on the mother-child dyad and aims to teach both the basic elements for effective childcare and behavioral strategies to cope with the difficulties that occur during this period.

## 2. Methods

### 2.1. Design of the Study

A randomized controlled field trial was carried out in two Italian hospitals of Rome, Italy. The CONSORT statement was followed to perform the research [33,34].

#### 2.1.1. Ethical Approval and Registration of the Protocol

Ethics approval for the full study was obtained (Protocol number 826/19, RIF.CE: 5559, date 12 September 2019). The protocol of the study was registered on the Clinicaltrials.gov database (accessed on 8 November 2021, ID number: 80209930587).

#### 2.1.2. Eligibility Criteria for Participants

The following eligibility criteria were applied: only women aged 18 years old or older and who were able to communicate in Italian were enrolled.

The following exclusion criteria were applied:

(1) women were excluded from the study if they or their babies had serious health problems;
(2) gestational age ≤37 weeks, weight of the baby <2500 g [35];
(3) APGAR score <7 immediately after birth [36].

Participants with whose criteria did not match those listed above were excluded from the study, as these might influence the outcomes [22,37] and pose a threat to the internal validity of the study.

Participants were recruited from the Obstetrics Units of the hospitals.

For organizational reasons, only mothers who live in the city of Rome were enrolled.

The enrollment of participants was conducted from 0 to three days postpartum by the researchers and research nurses using a brochure explaining the aim of the study.

The recruitment period was three months (October–December 2019). Prior to study participation, all women who agreed to participate were asked to sign a written consent form and to provide a contact phone number and e-mail.

#### 2.1.3. Randomization and Blinding

After obtaining the informed consent, the mothers were divided into two groups: the “individual intervention group”, called “IG”, and the “control group”, called “CG”. The CG received routine care only. This care involved postnatal support by nurses and midwives in the hospital and a follow-up (around one to six weeks post-delivery) via an outpatient appointment with the doctor at the hospital.

All women recruited were randomly allocated to the groups. Simple randomization was realized using a random number sequence generated with Epicalc 2000. For equal allocation to the two groups, odd and even numbers were used to indicate treatments I and C, respectively. The groups were matched according to the following variables:

1. age (>34 years, 34 is the mean age of Italian women at childbirth) [38];
2. vaginal delivery (Yes/No).

These variables are considered in the randomization because the literature has highlighted a causal link with stress levels [38,39].

Four groups were created for allocation: age >34 and vaginal delivery; age >34 and no vaginal delivery; age ≤34 and vaginal delivery; age ≤34 and no vaginal delivery. The random number was associated to one group on the basis of the residue class modulo 4: the least residue system modulo 4 is {0, 1, 2, 3}.

### 2.2. Data Collection

The recruitment lasted two weeks. During the delivery, a researcher requested that each participant sign consent forms and then collected the demographic information for matching and performing the randomization.

During this preliminary phase, a unique code was assigned to each woman.

For organizational reasons, one researcher had a paper sheet that reported the codes associated to the women’s names. After the recruitment and the randomization phase a data collection phase was started.

A message that contains the link to the questionnaire and the personal code was sent by phone. The personal code was used to identify the on-line questionnaires and preserve the anonymity of the participants.

The questionnaire was created using Google Forms and administered online four times:

1. At T0: about one week after the hospital delivery;
2. At T1: about one months after the delivery and after the home intervention;
3. At T2: three months after the delivery;
4. At T3: six months after the delivery.

### 2.3. Questionnaire

An online questionnaire was used to obtain socio-demographic data including age, civil status (single or not), employment (student/worker/no worker), educational level (middle school/high school/university), ethnicity, *the birth date*, primiparous (yes/no), the number of children living at home, and age, vaginal birth (yes/no), and characteristics of breastfeeding practice.

The questionnaire adopted for data collection was composed by three validated scales:

1. The Karitane Parenting Confidence Scale (KPCS) [40] was used. More precisely, the Italian version (KPCS-IT) validated by Mannocci et al. was included [41]. The KPCS scale measures perceived parental self-efficacy (PPSE), which is defined as ‘‘beliefs or judgments a parent holds of their capabilities to organize and execute a set of tasks related to parenting a child’’ [42]. The 15-item scale, based on self-efficacy theory [15], was developed to assess the PPSE of parents with infants aged 0–12 months. The factor analysis has revealed a three-factor structure: efficacy, support, and child development. This 15-item questionnaire was scored on a five-point Likert scale, where 0 = No, hardly ever; 1 = No, not very often; 2 = Yes, some of the time; 3 = Yes, most of the time). The internal consistency of the questionnaires KPCS-IT was estimated as 0.801 [41].
2. The Parental Stress Scale (PSS) [42] was used; more precisely, the Italian version (PSS-IT) validated by Mannocci et al. [41]. The PSS scale consisted of 18 items rated on a 5-point Likert scale (1 = low agree/5 = strong agree) The total score was obtained by summing up the value for each item. A higher score indicates a higher level of parental stress. The internal consistency of the PSS-IT studied by Mannocci et al. reported a Cronbach’s alpha = 0.862 [41].
3. The Italian version of the Edinburgh Postnatal Depression Scale (EPDS) [43,44,45]. The EPDS version published by Benvenuti et al. [43] is used to measure maternal depressive symptoms. The EPDS is a self-report screening measure used to detect symptoms of postpartum depression. Scores >12 on the EPDS are correlated with a diagnosis of major depressive disorder (MDD) [46].

During the administration of the follow-up questionnaires (T1, T2 and T3), the section on socio-demographic variables was removed.

### 2.4. HAPPY MAMA Intervention

#### 2.4.1. Personnel Involved in the Intervention

The health care workers involved in the administration of the interventions consisted of midwives, nurses, job-infant care workers and students in obstetrics who acted as tutors during the interventions.

#### 2.4.2. Intervention

A training course was carried out by a childcare worker and a midwife with high experience in childcare and home interventions. The participants in the training course were the operators that were involved in the administration of the intervention for the “IG”. The HAPPY MAMA intervention includes educational and mindfulness training and simulations of typical events. Given the importance of communication skills training and better outcomes in studies where skills practice has taken place, the interventionists developed their skills through patient simulations and role-play scenarios with one another and with the facilitators before interacting with study participants.

Objectives

The objective of the HAPPY MAMA intervention is to improve the maternal self-efficacy and mood control. In other words, it is to offer supports and techniques to increase confidence and to reduce stress. In particular, the first goal is to make new mothers recognize their abilities and to increase self-awareness: control and self-management are important for the process of learning mindfulness and with regard to the ability to care for the baby; the second one is to put new mothers in a condition to implement strategies and techniques practically in order to pursue goals to restore mental well-being.

Structure

The HAPPY MAMA intervention finds analogies and inspirations in problem-solving training [47]. The problem solving training is a therapeutic intervention, used if the gambler shows poor problem solving skills when coping with excessive gambling activities. A therapist usually introduces a problem solving technique [48] that involves the following five steps: (i) defining the problem, (ii) collecting information about the problem, (iii) generating different solutions, (iv) listing advantages and disadvantages for each solution, and (v) implementing and evaluating the solution. According to this approach, the intervention was thought and planned as follows:

1. Listening and establishing relationship phases

The first step is characterized by listening and understanding the critical points from the new mother.

This requires the use of listening skills, empathy, authenticity, and acceptance. The operator maintains a nonjudgmental approach and allows the woman to determine the need for behavioral change, rather than offering unsolicited advice on the need for change.

2. Analysis of the problems

The situation has to be carefully evaluated, considering the discomfort and emotional distress involved. The stress situation is described in a subjective way, from the new mother, and she will assign a grade of discomfort for each problem.

3. Assessment

The operator will carry out a multidimensional evaluation of the mother within the dyad. The operator will evaluate the strategies implemented by the new mother to face problems and difficulties, for example: how she routinely handles organizational problems, how she experiences breastfeeding if there is a lack of sleep, and how she considers her family and support network.

The evaluation will have to consider the environment as a whole, with attention to facilitators and barriers.

4. Definition of the problem and the goal of the intervention

The problems detected by the operator may be explained and summarized to the participants. The operator only explores ways to implement change once the woman expresses the desire and confidence to change.

The shared identification of the mothers’ priority will lead to the definition of a tailored plan aimed at achieving specific goals such as the reduction of the stress levels, the reduction in sleep deprivation (hours of sleep per night), optimization of breastfeeding (number, duration and quality), and increased well-being (mental health, physical health).

Strategies of concrete action and planned behavior have to be adapted to the context and to the mother’s coping style.

The length of the intervention is about three hours in one day.

#### 2.4.3. Sample Size

The method of setting the pilot trial sample size was applied [49,50], and the following parameters were chosen to establish the sample size:

1. average depression score measured with EPDS after childbirth is equal to mean = 5.1 and SD = 2.96 [43];
2. hypothesis: SD is similar in the “IG” and the standardized difference (effect size) of EPDS will be 0.1 ≤ d ≤0.3 (small effect) [49], namely that the EPDS mean in the IG was lower 4.2 ≤ mean EPDS ≤ 4.8. The hypothesis of a small effect size was chosen because it is the first time that the HAPPY MAMA intervention was carried out and the effects are unknown. The small effect observed in the literature for other similar interventions was also considered [27,51];
3. the level of significance and power of the study are 95% and 80%, respectively.

On account of these parameters, the pilot sample size is *N* = 20 for each group.

An increment of 20% for possible missing data and lost to the follow-up was considered. A total of 48 women (24 women for each group), were recruited.

### 2.5. Statistical Methods

Pre-protocol analysis was adopted: the sample included only those patients who completed the treatment originally allocated at different times.

Descriptive statistics was used to show the characteristics of the sample and to analyze the feasibility and acceptability of the study protocol. Measures of central tendency (mean and median) and variability (Standard Deviation, SD, and minimum and maximum) for continuous variables and frequencies with percentages for categorical variables were computed.

The outcomes PSS, KPCS, and EPDS were described, stratifying by demographic characteristics and monitored in time periods (T0, T1, T2 and T3).

A univariate analysis was conducted to compare the different groups (IG and CG) versus primary (PSS and KPCS) and secondary outcomes (EPDS): non-parametric tests were applied to assess the possible differences of the scores between the two groups; a Chi-square test was used to determine possible independence between the groups versus categorical variables.

The tests for paired samples were used to assess the possible changes of the stress score during the follow-up of the control and intervention groups (T0 versus T1, T2 and T3).

Moreover, a bivariate analysis was conducted to assess the possible relationship between the three different outcomes. The correlation coefficient was computed using Spearman’s coefficient.

The reliability of the questionnaire was assessment by computing the Cronbach’s alpha coefficient.

The significance level was set at *p* < 0.05. Data was analyzed using the IBM SPSS Statistics version 25.0 for Windows (SPSS Inc., Chicago, IL, USA).

## 3. Results

Ninety-one women were considered to be eligible candidates for the study. Forty women refused to participate (response rate of 56%). The study population counted 51 mothers who answered questionnaires at different follow-up periods.

**Figure 1:**
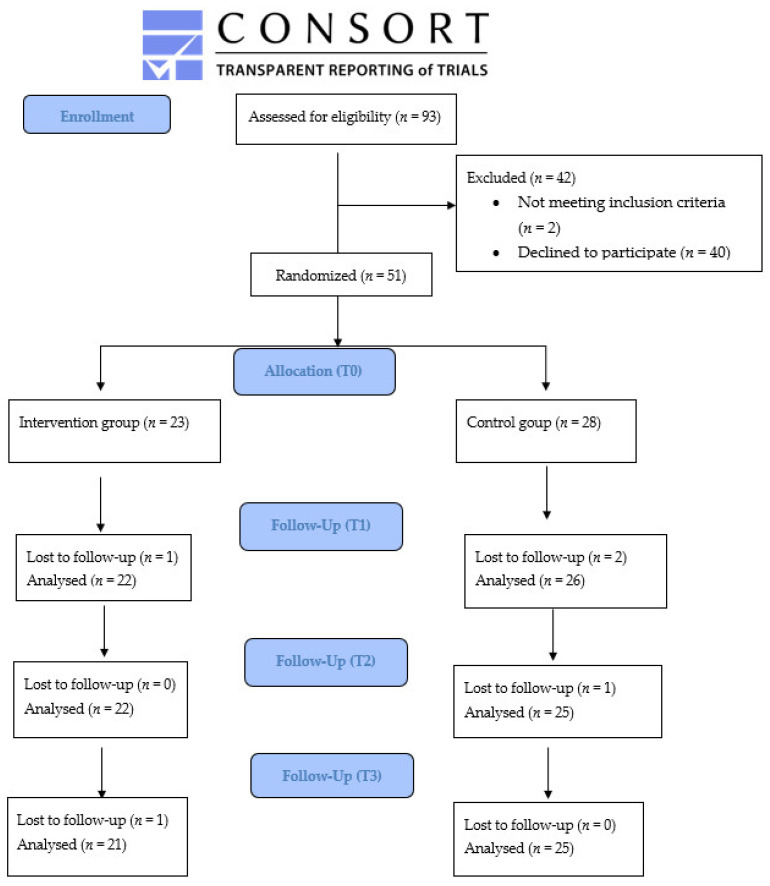
CONSORT flow diagram of the trial.

Concerning the group that refused to enter into the trial, it was possible to collect data on age, type of delivery, and civil status. The mean age was not different to the enrolled sample (*p* > 0.05). Half of these women had a vaginal birth and all of them live with a partner enrolled group (*p* > 0.05). The CONSORT flow diagram of the study enrolment is shown in Figure 1.

A total of 28 women were enrolled in the “CG” and 23 women were enrolled in the “IG”.

The mothers’ characteristics are described in Table 1. The mean age of the sample was 34.3 years old, with a range of 24–44 years old. The majority were graduates, workers, and first-time mothers. All mothers lived with their partners. As for the pregnancy experience, two-thirds of participants had a vaginal birth, according to the national data [52]; women received a different kind of support both during pregnancy and after birth, involving different health care professionals. Mothers adopted various kinds of the newborn feeding, with a high percentage of “Partial breastfeeding” (76.4%), which included both breast milk and formula milk.

The univariate analysis is described in Table 2. The comparison of the outcomes between the two groups in the different follow-ups was reported.

### 3.1. KPCS Score

The KPCS score progressively increased in the follow-ups in both groups. A low PPSE (KPCS score < 39) [38] was found at T0 for both groups and at T1 for “CG”.

KPCS scores showed a statistically significant difference in the two groups (*p* = 0.039), tat T1 (one month after the individual intervention).

Concerning the reliability of the questionnaire, the Cronbach’s alpha at T0 was 0.851.

### 3.2. PSS Score

The PSS score has generally been reduced at the end of the follow-ups. The “CG” mean ranged from 30.1(SD = 6.2) at T3 to 32.9 (SD = 9.1) at T1; while the “IG” mean value ranged from 34.3 (SD = 7.5) at T0 to 27.7 (SD = 5.6) at T1. There was not a recommended cut-off (see “Methods” paragraph), but higher scores correlated with higher perceived levels of stress. PSS scores showed a statistically significant difference in the two groups (*p* = 0.024) at two months after birth (T1).

Concerning the reliability of the questionnaire, the Cronbach’s alpha at T0 was 0.650.

### 3.3. EPDS Score

The EPDS score progressively decreased during the follow-ups in both groups, and the recommended cut-off (<12) was reported at all times in both groups.

EPDS scores showed no significant difference (*p* > 0.05) between the two groups in the follow-up.

With regard to the reliability of the questionnaire, the Cronbach’s alpha at T0 was 0.754.

The correlation analysis showed an inverse significant correlation between KPCS and PSS over all time: r = −0.464 (*p* = 0.001) at T0, r = −0.621 (*p* = 0.000) at T1, r = −0.598 (*p* = 0.000) at T2 and r = −0.474 (*p* = 0.000) at T3.

## 4. Discussion

The HAPPY MAMA Project results confirmed the assumption that the postpartum period is one of transition, and new mothers need time to adjust to their new role [53]; PSS, KPCS and EDPS scores improved in the follow-up periods, especially from two to six months postpartum, according to similar evidence [54]. The early postnatal period is characterized by lower maternal confidence (KPCS score < 39), and higher perceived stress (PSS score) and depressive symptoms. It is important to underline that the mentioned scores do not have the purpose of making any particular clinical diagnosis, as the questionnaires were self-administered

Several factors such as changing habits and lack of experience can affect either maternal confidence or mood; these feelings may improve after some weeks, after becoming more skilled and self-confident, as underlined by Kristensen et al. in a similar study [54]. This is also pointed out in the first phase of the HAPPY MAMA Project [41], which shows a strong inverse correlation between KPCS and PSS scores. The review carried out by Alberese et al. also showed a statistically significant inverse correlation between the perceived self-efficacy (PSE) and depression symptoms (*p* < 0.05), therefore at each follow up a better maternal PSE corresponds to lower perceived maternal stress [55]. Concerning the hypothesis of causal connection, that is that the PSE should buffer against stress levels, Albanese et al.’s review confirms that PSE is a key factor affecting both the parent and the child’s well-being. On the other hand, reverse causality could be likely too: parents who are more stressed have less resources to cope with their child. Consequently, their parenting is less effective and hence, their parenting self-efficacy is diminished. This alternative interpretation should be in line with several theoretical accounts like the Family Stress Model [56]. In order to understand if there is a causal relationship, it will be important for future longitudinal works to aid in clarifying the directionality of the relationships between PSE and key outcomes, as well as to monitor how these relationships function over time and in varying contexts.

The present study showed a statically significant difference between the PSS and KPCS score of the “IG” and “CG” only at the first follow-up (T1): “I” group showed a higher KPCS score (Median = 41.0) than “C” group (Median = 37.5) two months after birth and one month after individual home intervention; “IG” also showed a lower PSS score (Median = 26.0) than “CG” (Median = 29.5). The HAPPY MAMA intervention is probably “effective” in the short term, although the definition of efficacy is sufficiently restrictive in this study. In fact, it should be possible to investigate other outcomes such as maternal sleep, baby sleep and weight gain. On the other hand, the results can suggest that a more structured or/and longer support (e.i. more than one home visit or telephone call, and/or counselling or educational booklets) in the intervention could be thought too. Since the intervention was proposed for the first time in this research, it is not possible to find very similar studies. Regardless, the results of this study are in line with those obtained in other studies in first-time mothers.

Missler et al. conducted a randomized controlled trial that was to examine the effectiveness of a brief psychoeducational intervention to prevent postpartum parenting stress, to decrease symptoms of depression and anxiety, and to enhance parental well-being and the quality of caregiving behavior [51]. The intervention consisted of a booklet, a video, a home visit, and a telephone call. The primary outcome was parenting stress postpartum measured using a different tool: the Parenting Stress Index [57].

No differences emerged in levels of parenting stress between the intervention and control group over time. Also, there was no effect of the intervention on symptoms of depression and anxiety, nor on the indices of parental wellbeing (satisfaction with the parenting role, self-efficacy, and sleep quality and quantity) [51]. These results are in agreement with the follow-up results of the HAPPY MAMA trial.

Another similar study was published by Shorey et al. [58]. They studied the effectiveness of a postnatal psychoeducation programme in enhancing maternal self-efficacy and social support and reducing postnatal depression among primiparous women. The intervention group received 90-min face-to-face educational sessions during the home visit, an educational booklet and three follow-up telephone calls. The authors used the same tool to measure the depression risk [45], but a different questionnaire for perceived maternal parental self-efficacy [59] was used. The stress level was not considered. Outcomes were measured at three time points: baseline (on the day of discharge between one to three days postpartum), at six weeks postpartum, and at 12 weeks postpartum. Their findings were shown to be effective in enhancing maternal parental self-efficacy and in reducing postnatal depression at six and 12 weeks postpartum.

In another Iranian clinical trial, the effect of pregnancy training classes based on Bandura’s self-efficacy theory on postpartum depression and anxiety was studied. In this case the findings showed that pregnancy training classes based on Bandura self-efficacy theory decreased depression and anxiety during the pregnancy and one month after the delivery [14,60]. The results at one month after the delivery showed a similar effect with the HAPPY MAMA intervention, though different outcomes and tools were applied and the training stared before the delivery.

In agreement with some reflections of Missler et al., several factors could have played a role here. First, it is possible that the intervention is effective on other measures that we did not take into account in this study (e.g., observed sensitive responsiveness, infant well-being). Second, the intervention might be more effective for specific groups of parents (i.e., the sample was relatively well-educated, reporting a high educational level). Also, we lack information on the participants’ psychosocial history. Finally, it is possible that the intervention has no added value [51].

The study presents several limitations. The first limitation was the different distribution of the educational level in the sample in comparison with the female adult Italian population: 80.4% of the sample were graduates, even if the percentage of Italian graduated women was 22.4% in 2019 [61]. Secondly, in this project the HAPPY MAMA intervention was applied in “healthy” babies and mothers and with a “normal” gestational age; the effect for other criteria of new-mothers could lead to different results.

With regard to the sample size, the results in this small sample must be considered carefully, since the study population cannot be considered representative of the Italian new mother population. This study, however, lays the foundation for a main trial and overall trial. Additionally, the pilot design of the study did not allow one to have a robust conclusion on the effect of the HAPPY MAMA intervention on the maternal self-efficacy at T1, because they might just result from luck due to the alpha error inflation. Regarding the definition and measurement of PSE, the literature identified a need for extensive psychometric evaluation in future work [62].

With regard to pregnancy, the mothers recruited received different supports during the pregnancy and postpartum period (public or private health care, clinicians involved), and this could have indirectly influenced primary outcomes. Although it was not possible from an ethical point of view to prohibit any other support required by women (especially in the “CG”), if needed.

Moreover, from a statistics point of view, the small sample size did not allow for the performing of a multivariate regression model. Consequently, it was not possible to evaluate the simultaneous effect of different independent variables.

Concerning the EPDS measurement, this study considered the international clinical EPDS cut-off >12 [46], while other studies used a clinical EPDS cut-off >8 [54] or >10 [63]. This choice could have underestimated the risk.

In addition, HAPPY MAMA was a one-time home intervention. It was carried out about one month after birth by self-employed health care providers unknown to mothers. Scientific evidence shows that new-mothers need a holistic support earlier than six-weeks postpartum, and one in the first 10 days would have been better [64]. Similarly, more than one visit would have been better, underlying the importance of the continuity of care to build trusting relationships [65,66,67]: better to talk about “trusted midwife” or “caseload midwifery” [68]. While previously a mother and her child spent five tosix days in the maternity ward, now they generally go home after one or two days; the health care system is not organised to keep pace with these changes [69]. Postnatal continuity care and postpartum home visits are important services that can improve maternal coping and confidence, empowering mothers to take care of their children’s health and theirs as well, thus preventing any psychological consequences [63]; that is why the World Health Organization (WHO) recommends “Midwife-led continuity-of-care models, in which a known midwife or small group of known midwives supports a woman throughout the antenatal, intrapartum and postnatal continuum” [68]. Finally, while absence of evidence is not evidence of absence, it should also acknowledge the possibility that one session of intervention aimed at providing information is not sufficient to influence parents’ levels of distress, wellbeing and their caregiving quality.

On this field a jeopardized situation of activities is present in Italy: in some Italian regions started to adapt health care services to ensure continuity of care through hospitals and territorial (counselling centers) protocols. In 2010, an institutional “state regions agreement” was created in order to support the services and activities for well-functioning midwifery programs [69,70].

Regarding the strengths of the study, the choice of the home visit has been received favorably by the new-mothers. The support offered to the IG group was never refused (no drop-outs) and was appreciated. In addition, the participation rate in the study (at T3) is acceptable in both groups (8% drop-out in IG and 10% in the CG). The pilot fixed sample size was respected.

The missing values in the questionnaire are absent.

## 5. Conclusions

This pilot experimental trial was inspired by the willingness to investigate the psychological wellness of new-mothers and to analyse the effect of a single midwifery intervention on mothers’ health. It showed that maternal confidence, reduction of stress and mood improve in the months ahead of the intervention. The maternal self-efficacy and perceived stress can be positively influenced by health care support, although this study shows mild effects of one-time intervention. There is therefore the need to analyse the topic in order to better highlight maternal needs and to identify which supports, combinations and how many sessions in an intervention ensure successful transition to motherhood. Large overall randomized trials are required to understand which are the most effective interventions in the postpartum period to sustain and increase women’s health and the mother-newborn dyad. It is recommended that further research show the effect of blending mindfulness and skills-based prenatal education program on self-efficacy. In fact, the ultimate goal is institute a system to support women after their delivery that is currently missing in Italy.

## Figures and Tables

**Table 1:** Characteristics of the sample.

Variables		Total (*N* = 51)	CG (*N* = 28)	IG (*N* = 23)
		*N* (%)	*N* (%)	*N* (%)
Educational level	Middle school	3 (5.9)	1 (3.6)	2 (8.7)
	High school	7 (13.7)	3 (10.7)	4 (17.4)
	University	41 (80.4)	24 (85.7)	17 (73.9)
Employment	Worker	42 (82.3)	26 (92.9)	16 (69.6)
	Housewife	2 (3.9)	0 (0.0)	2 (8.7)
	Student	1 (2.0)	0 (0.0)	1 (4.3)
	No worker	6 (11.8)	2 (7.1)	4 (17.4)
Number of children	1	34 (66.7)	18 (64.3)	16 (69.6)
	2	13 (25.5)	9 (32.1)	4 (17.4)
	>2	4 (7.8)	1 (3.6)	3 (13.0)
Type of birth	Vaginal birth	37 (72.5)	22 (78.6)	15 (65.2)
	Caesarean section	14 (27.5)	6 (21.4)	8 (34.8)
Support received during pregnancy	No	10 (19.6)	6 (21.4)	4 (17.4)
	Yes (Hospital/ASL)	37 (72.6)	19 (67.9)	18 (78.3)
	Yes (Private)	4 (7.8)	3 (10.7)	1 (4.3)
Visits/counselling post-partum	No	33 (64.7)	19 (67.9)	14 (60.9)
	Midwife/childcare	13 (25.5)	7 (25.0)	6 (26.1)
	Clinician	5 (9.8)	2 (7.1)	3 (13.0)
Kind of breastfeeding	Exclusive	11 (21.6)	6 (21.4)	5 (21.7)
	Partial	39 (76.4)	22 (78.6)	17 (74.0)
	No (bottle)	1 (2.0)	0 (0.0)	1 (4.3)
Number of feedings	4–5/day	2 (3.9)	1 (3.6)	1 (4.3)
	6–8/day	31 (60.8)	18 (64.3)	13 (56.6)
	9–10/day	13 (25.5)	6 (21.4)	7 (30.4)
	>10/day	5 (9.8)	3 (10.7)	2 (8.7)

CG: Control Group; IG: Intervention Group; ASL: Azienda Sanitaria Locale (Local Health Unit).

**Table 2:** Univariate analysis for the comparison of the KPCS, PSS and EPDS scores between the groups (CG and IG) in the different times of the follow-up.

Variables	(Follow-Up)	CG	IG	*p* *
		Mean ± SD Median (Min-Max)	Mean ± SD Median (Min-Max)	
KPCS	(T0)	35.8 ± 6.0 36.5 (15.0–45.0)	35.0 ± 5.8 35.0 (24.0–44.0)	0.544
	(T1)	37.0 ± 4.9 37.5 (27.0–44.0)	39.7 ± 4.2 41.0 (32.0–45.0)	0.039
	(T2)	39.3 ± 3.6 40.0 (32.0–44.0)	39.0 ± 5.6 41.0 (22.0–45.0)	0.614
	(T3)	39.7 ± 4.2 41.0 (28.0–45.0)	40.5 ± 3.8 41.0 (32.0–45.0)	0.458
PSS	(T0)	31.1 ± 6.2 30.0 (21.0–45.0)	34.3 ± 7.5 33.0 (24.0–50.0)	0.105
	(T1)	32.9 ± 9.1 29.5 (22.0–55.0)	27.7 ± 5.6 26.0 (20.0–39.0)	0.024
	(T2)	31.1 ± 7.7 30.5 (20.0–51.0)	31.3 ± 9.7 30.0 (18.0–52.0)	0.864
	(T3)	30.1 ± 8.9 28.5 (19.0–54.0)	30.0 ± 8.8 28.0 (18.0–48.0)	0.894
EPDS	(T0)	8.4 ± 4.1 8.0 (0.0–19.0)	8.0 ± 3.2 8.0 (1.0–16.0)	0.924
	(T1)	7.5 ± 4.1 8.0 (0.0–15.0)	6.3 ± 3.5 7.0 (0.0–12.0)	0.246
	(T2)	6.9 ± 3.5 7.0 (1.0–14.0)	6.2 ± 4.2 6.0 (0.0–13.0)	0.575
	(T3)	6.6 ± 5.0 7.0 (0.0–15.0)	6.0 ± 4.8 7.0 (0.0–16.0)	0.575

T0 = one week after delivery; T1 = one month; T2 = three months; T3 = six months. * *p*-value of Mann-Whitney test. Bold *p* < 0.05. CG: Control Group; IG: Intervention Group; KPCS: Karitane Parenting Confidence Scale; PSS: Parental Stress Scale; EPDS: Edinburgh Postnatal Depression Scale.

## Data Availability

The datasets used and/or analysed in the current study are available from the corresponding author on reasonable request.
